# The Potential Role of Presepsin in Predicting Severe Infection in Patients with Diabetic Foot Ulcers

**DOI:** 10.3390/jcm13082311

**Published:** 2024-04-17

**Authors:** Eun Yeong Ha, Il Rae Park, Seung Min Chung, Young Nam Roh, Chul Hyun Park, Tae-Gon Kim, Woong Kim, Jun Sung Moon

**Affiliations:** 1 Department of Internal Medicine, Yeungnam University Medical Center, Daegu 42415, Republic of Korea; ghkies@ynu.ac.kr (E.Y.H.); pil0411@naver.com (I.R.P.); smchung@ynu.ac.kr (S.M.C.); woongwa@yu.ac.kr (W.K.); 2 Department of Surgery, Yeungnam University Medical Center, Daegu 42415, Republic of Korea; nyn0913@gmail.com; 3 Department of Orthopedic Surgery, Yeungnam University Medical Center, Daegu 42415, Republic of Korea; chpark77@yu.ac.kr; 4 Department of Plastic Surgery, Yeungnam University Medical Center, Daegu 42415, Republic of Korea; kimtg0919@yu.ac.kr

**Keywords:** diabetic foot, foot ulcer, presepsin, CD14, amputation

## Abstract

**Background/Objectives**: Diabetic foot ulcers are one of the complications in patients with diabetes, which can be caused by infection, neuropathy, and blood vessel disorder. Among them, infection is the most common cause, and if it becomes worse, amputation may be necessary. So, it is important to detect and treat infections early, and determining indicators that can confirm infection is also important. Known infection markers include white blood cells (WBCs), the erythrocyte sediment rate (ESR), C-reactive protein (CRP), and procalcitonin, but they are not specific to diabetic foot ulcers. Presepsin, also known as soluble CD14, is known to be an early indicator of sepsis. Recent studies have reported that presepsin can be used as an early indicator of infection. This study investigated whether presepsin could be used as an early marker of severe infection in patients with diabetic foot ulcers. **Methods:** We retrospectively studied 73 patients who were treated for diabetic foot ulcerations from January 2021 to June 2023 at Yeungnam University Hospital. **Results:** Out of a total of 73 patients, 46 patients underwent amputations with severe infections, and the WBC level, ESR, and CRP, procalcitonin, and presepsin levels were significantly higher in the group of patients who underwent amputations. The cutoff of presepsin, which can predict serious infections that need amputation, was 675 ng/mL. A regression analysis confirmed that presepsin, HbA1c, and osteomyelitis significantly increased the risk of severe infections requiring amputation. **Conclusions:** Presepsin will be available as an early predictor of patients with severe infections requiring amputations for diabetic foot ulcerations.

## 1. Introduction

Diabetes mellitus is one of the metabolic diseases characterized by hyperglycemia resulting from defects in insulin secretion, insulin function, or both [1]. Diabetes mellitus is a complex, chronic condition requiring continuous medical care with multifactorial risk reduction strategies beyond glycemic management [2]. In 2021, 38.4 million Americans, or 11.6% of the population, had diabetes. Of the 38.4 million adults with diabetes, 29.7 million were diagnosed, and 8.7 million were undiagnosed in the US [3]. According to the data released by the Korean Diabetes Association in 2021, there were 6.05 million adults aged 30 or older with diabetes, and adults aged 65 or older accounted for 39.2% of all the patients with diabetes in Korea [4]. The chronic hyperglycemia of diabetes is associated with long-term damage, dysfunction, and failure of various organs [1]. Chronic exposure to a glucose-rich environment creates physiological and pathophysiological changes. There are several pathways by which hyperglycemia exacerbates its toxic effect on cells, tissues, and organ systems. Numerous complications have been associated with hyperglycemia. Dysregulation in the cardiovascular system, along with nephropathy, retinopathy, neuropathy, and diabetic foot ulcers, may arise in the advanced stages of diabetes. A high glucose level potentiates a suitable environment for infections [5]. A diabetic foot ulceration is a significant complication in an individual with diabetes, as initial foot wounds progress to ulcers, posing serious concerns in patients with diabetes. The lifetime risk of developing a diabetic foot ulcer ranges from 19% to 34% [6]. The main causes encompass infection, blood circulation disorder, and neurological disorders, with infection occurring in about 50% to 60% of diabetic foot ulcers. Particularly, when the infection progresses to the bone or joint, surgical treatment may be required, potentially involving a procedure like amputation.

When infection is suspected, it is advisable to perform tests for inflammatory markers and wound culture with representative inflammatory markers, including C-reactive protein (CRP), procalcitonin, and presepsin. CRP is an acute phase protein synthesized by the liver, characterized by high sensitivity but a low specificity for bacterial infection. CRP is significantly higher in patients with diabetic foot ulcers, but it is known to be diagnostically more effective with procalcitonin rather than CRP alone [7,8]. Procalcitonin is used for assessing sepsis and infection, although it has limitations and may yield false positive results in multiple traumas [9]. Procalcitonin is significantly higher in infected diabetic foot ulcers, and compared to CRP, procalcitonin has higher sensitivity and specificity in infected diabetic feet [10,11]. However, while studies have shown that procalcitonin is significantly higher in patients with osteomyelitis, there is also no significant difference between an amputation group and non-amputation group regarding diabetic feet. The predictive role of procalcitonin may be less effective in a surgery group [12].

As a glycoprotein expressed in the membranes of monocytes and macrophages, CD14 functions predominantly as a co-receptor of the lipopolysaccharide–lipopolysaccharide binding protein complexes, inducing the inflammatory cascade [13]. Consequently, during the inflammatory reaction, presepsin is formed by the cleavage of the N-terminal of soluble CD14, a member of the Toll-like receptor family [14]. Presepsin is known as a diagnostic and prognostic biomarker in sepsis [15]; it can particularly help with the early diagnosis of sepsis and septic shock, with its levels correlating with severity [13,16,17]. Several studies have demonstrated an elevation in plasma presepsin levels during bacterial sepsis, which subsequently decline in response to appropriate therapy. Notably, its sensitivity and specificity have been found to be comparable to those of procalcitonin [18,19]. Furthermore, presepsin remains unaffected by severe trauma or severe burns [20]. Another study has suggested the potential utility of presepsin as an early indicator of infectious disease [21].

This study investigated the diagnostic and prognostic roles of presepsin in patients with diabetic foot ulcers, especially those without sepsis.

## 2. Materials and Methods

### 2.1. Study Population

This retrospective, case–control study included patients with diabetic foot ulcers who were admitted to Yeungnam University Hospital, Daegu, Republic of Korea from January 2021 to June 2023. This study was conducted through access to medical records; initially, 194 patients were selected after being examined for eligibility. The inclusion criteria were as follows: patients (1) aged > 18 years (2) who were diagnosed with diabetic foot ulcers and (3) underwent a presepsin test at the time of hospitalization. Exclusion criteria included patients who (1) previously had a diabetic foot amputation on the same side and (2) had a history of sepsis under the sepsis-3 criteria. The criteria suggest that patients with at least two of the three clinical variables can be classified as having sepsis. The variables were as follows: (1) a low blood pressure (systolic blood pressure ≤ 100 mgHg), (2) a high respiratory rate (≥22 breaths per minute), or (3) altered mentation (Glasgow coma scale < 15) [22]. A total of 73 patients were included in the final analysis. The study protocol adhered to the tenets of the Declaration of Helsinki and was approved by the Institutional Review Board of Yeungnam University Hospital (approval no. 2023-10-054). The Ethical Committee approval date is 3 November 2023.

### 2.2. Clinical and Biochemical Measurements

Body mass index (BMI) was calculated as the square of the weight division height. Estimated glomerular filtration rate (eGFR) was calculated using the Modification of Diet in Renal Disease (MDRD) equation = 186 × (serum creatinine)^−1.154^ × (years)^−0.203^ (×0.742 if female) [23]. Ankle brachial index (ABI) and toe brachial index (TBI) measured blood flow and blood pressure at each part by winding blood pressure cuffs for both arms and legs (MultiLab Series II LHS, Unetixs Vascular, Inc., Orlando, FL, USA). The diagnostic criteria for osteomyelitis were as follows: (1) previous diagnosis by a doctor (2) read as osteomyelitis in imaging tests or (3) diagnosed with osteomyelitis after surgery. Wound culture was tested by collecting samples from diabetic foot ulcer.

The diagnostic criteria for diabetes mellitus were as follows: (1) previous diagnosis by a doctor, (2) taking diabetic medication, or (3) having a fasting glucose of ≥126 mg/dL and glycated hemoglobin (HbA1c) of ≥6.5% [24].

Records of smoking and drinking history were checked at the time of admission. The diagnostic criteria for hypertension were as follows: (1) previous diagnosis by a doctor and (2) taking hypertensive medication. Records were used to confirm whether the patient was undergoing hemodialysis or peritoneal dialysis. Records were used for diabetic retinopathy, including non-proliferative retinopathy or proliferative retinopathy. The diagnostic criteria for diabetic neuropathy were as follows: (1) previous diagnosis by a doctor and (2) taking neuropathy drugs. Records were used for stroke, such as brain hemorrhage or brain infarction. Records were also used for coronary artery disease, including angina or myocardial infarction.

The initial assessment of diabetic foot ulcer was conducted using the staging system of the University of Texas [25]. Grade 0 represents a pre- or post-ulcerative lesion that is completely epithelialized. Grade I indicates a superficial ulcer without the involvement of a tendon capsule or bone. Grade II refers to ulcer penetrating to tendon or capsule. Grade III indicates an ulcer penetrating to the bone or joint. Stages are categorized as A (neither infection nor ischemia), B (with infection), C (with ischemia), and D (with infection and ischemia).

The X-ray of the diabetic foot’s laterolateral and anteroposterior view was taken. The five vascular sites were as follows: (1) dorsalis pedis artery from the ankle joint to its disappearance inside the metatarsal bones; (2) lateral plantar artery from the bifurcation of the posterior tibial artery to the visible part of the distal plantar arch; (3) first metatarsal artery from its proximal origin to the metatarsal–phalangeal joint; (4) first toe artery; (5) other toe arteries from the metatarsal–phalangeal joint line to the tips of the toes. Medial arterial calcification (MAC) score was evaluated with a total of 5 points, with 1 point each for calcification of 2 cm or more in the dorsalis pedis artery, lateral plantar artery, and first metatarsal artery, and 1 point each for calcification of 1 cm or more in the first toe artery and other toe arteries. A score of 0–1 points was classified as no, a score of 2–3 points was classified as moderate, and a score of 4–5 points was classified as severe [26].

Venous samples for presepsin were collected from the patients’ antecubital veins, placed into EDTA tubes, and transported. The analysis was performed within 4 h of blood collection. The samples were mixed with magnetic particles coated with anti-presepsin polyclonal antibodies, and anti-presepsin monoclonal antibodies labeled with alkaline phosphatase (PATHFAST Presepsin, NIPRO Corporation, Odate, Japan) were mixed with the samples [27]. The presepsin present in the sample was mixed with anti-presepsin antibodies to form immune complexes with enzyme-labeled antibodies and antibody-coated particles. After removing the unbound antibody, chemiluminescent substrates were added to the immune complex, and the chemiluminescence produced by the enzyme reaction was detected. The normal range of presepsin is less than 300 pg/mL, and presepsin was defined as increasing when it was 300 or more.

Amputation included both major amputation (above ankle) and minor amputation (below ankle).

### 2.3. Statistical Analysis

All statistical tests were performed using SPSS software (version 21, IBM Inc., Chicago, IL, USA). Baseline characteristics were expressed as mean ± standard deviation for continuous variables and as numbers and percentages for categorial variables. Differences between two groups were assessed using the Mann–Whitney U test for continuous variables and chi-square test for categorial variables. Differences among four groups were assessed using the Kruskal–Wallis H test. Logistic regression analysis was used to conduct a risk factor assessment of the need for diabetic foot ulcer amputation. The odds ratio (OR) was reported with 95% confidence interval (CI). The Receiver Operation Characteristic Curve (ROC curve) was used to assess presepsin to predict amputation. Statistical significance was set at *p* < 0.05.

### 2.4. Outcomes

The first outcome was the difference between the group with severe infection requiring amputation and the no amputation group, particularly regarding the level of inflammatory markers. The second outcome was the cutoff of inflammatory markers predicting serious infection with a need for amputation, and the difference between high presepsin group and low presepsin group. The third outcome was the risk factor for severe infection that required amputation in patients with diabetic foot ulcers.

## 3. Results

A total of 73 patients were hospitalized for diabetic foot ulcers, of which 46 patients underwent amputations (Table 1). There was no significant difference in age between the two groups. In the amputation group, the proportion of men was as high as 69.6%. Additionally, this group showed a significantly longer hospitalization period at 32.4 days, and the rate of osteomyelitis was significantly higher at 78.3%. Furthermore, the blood urea nitrogen (BUN) and creatine levels were higher in the amputation group, and the eGFR was lower, although significant differences were not observed between the two groups.

**Figure 1:**
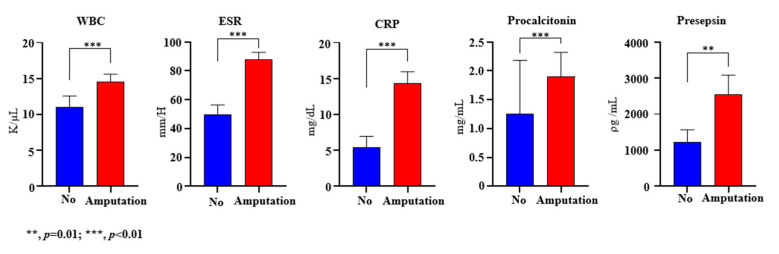
Baseline inflammatory markers.

The WBC level was significantly higher in the amputation group compared to the no resection group (14.6 ± 7.1 vs. 11.0 ± 8.3, *p* < 0.01) (Figure 1). The ESR was also significantly elevated in the amputation group (87.6 ± 34.8 vs. 49.5 ± 35.5, *p* < 0.01). The CRP level was significantly elevated in the resection group (14.4 ± 10.8 vs. 5.5 ± 7.9, *p* < 0.01). Procalcitonin was also significantly higher in the same group (1.9 ± 2.8 vs. 1.3 ± 4.8, *p* < 0.01). The presepsin levels were also significantly higher in the amputation group compared to the no resection group (2552.2 ± 3611.8 vs. 1215.9 ± 1814.1, *p* = 0.01).

**Figure 2:**
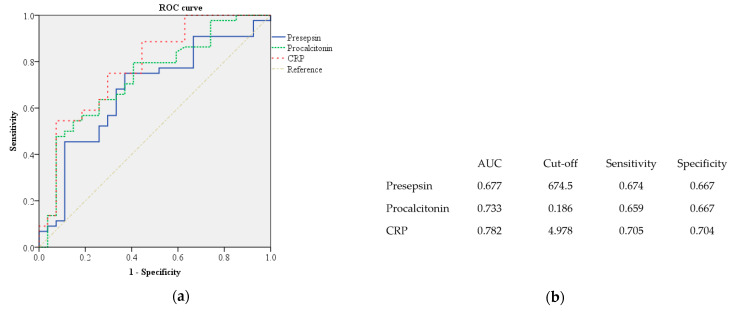
(**a**) ROC curve of presepsin for predicting amputation. (**b**) Area under curve, cutoff value, sensitivity, and specificity of presepsin, procalcitonin, and CRP.

In the ROC curve analysis, the cutoff value of presepsin for predicting amputation was 674.5 (area under curve (AUC) = 0.677, sensitivity = 0.674, specificity = 0.667), and a presepsin concentration of 675 or higher is considered to be indicative of the need for amputation (Figure 2). The cutoff value of procalcitonin for predicting amputation is 0.186 (AUC = 0.733, sensitivity = 0.659, specificity = 0.667), and that of CRP is 4.978 (AUC = 0.782, sensitivity = 0.705, specificity = 0.704).

All patients were classified into two groups according to the presepsin levels, which could predict severe infection requiring amputation (Table 2). There were 33 patients with a presepsin level below 675 pg/mL, while 40 patients had a presepsin level of 675 pg/mL or more. The mean age was significantly lower in the group with a higher presepsin level. The BMI was significantly higher in the group with a high presepsin level, and the duration of diabetes was significantly shorter. The higher the presepsin level, the longer the hospital stays, and the higher the presepsin level, the higher the average procalcitonin, WBC level, ESR, and CRP. However, there was no difference in the rate of bacterial identification according to the presepsin level when the wound culture test was performed. The eGFR was significantly lower in the group with a high presepsin level. The higher the presepsin level, the higher the proportion of patients who underwent minor or major amputation.

In a logistic regression analysis that was performed to identify the risk factors of severe infection requiring amputation in patients with a diabetic foot, the risk decreased with the WBC (Table 3). The risk of severe infection needing amputation was not significantly increased according to age and the ESR. According to the presepsin group, the risk was significantly increased in the group with a presepsin level of 675 or more. As the glycated hemoglobin increased, the risk of severe infection that required amputation increased by 1.8 times. The risk was also significantly increased when there was a finding of osteomyelitis.

## 4. Discussion

Patients with a diabetic foot who underwent amputation showed a higher prevalence of osteomyelitis and elevated inflammatory markers, including WBC, ESR, CRP, procalcitonin, and presepsin. In the patient group with a presepsin level of 675 or more, the proportion of patients who underwent minor or major amputation was high, and the duration of hospital stay was long. In this group, the levels of WBC, ESR, CRP, and procalcitonin were significantly high, and the eGFR was low. However, there was no significant difference in the rate of osteomyelitis according to the presepsin level. After adjusting for variable clinical factors, HbA1c, osteomyelitis, and a presepsin level of 675 or more were found to be closely associated with severe infection requiring amputation.

The WBC level, ESR, CRP level, and procalcitonin level are biomarkers for diabetic foot infections [10,12,28,29,30]. The CRP and procalcitonin levels were effective markers for discriminating against diabetic foot infections, but the WBC level was not predictive of diabetic foot infections [8]. In the current study, the WBC level, ESR, and CRP, procalcitonin, and presepsin levels were all significantly higher in the group with severe infections requiring amputation.

In addition, when presepsin was divided into two groups and compared between groups, it was showed that as the level increased, the WBC level, ESR, CRP level, and procalcitonin level also increased. After adjustments, HbA1c, osteomyelitis, and presepsin ≥ 675 pg/mL significantly increased the risk of severe infections needing amputation, whereas the WBC level decreased the risk of amputation, which should be interpreted with caution. The WBC level is one of the most used metrics to investigate infection. But sepsis or severe infection may cause either leukocytosis or leukopenia. A lot of septic patients exist between these two extremes, with a normal WBC range [31]. It may also be difficult to use the WBC level as an indicator of severe infection in patients with diabetic foot ulcers. The ESR was also shown to be a risk factor for severe infection requiring amputation, but it did not significantly increase it. It is known that the ESR is nonspecific to sepsis or severe infection, and it also increases in inflammatory disease [32]. Though the CRP and procalcitonin levels were not significantly independent risk factors for severe infection requiring amputation, their diagnostic accuracy for amputation was fair (0.7 ≤ AUC < 0.8). Taken together, integrated interpretations of inflammatory indicators will be helpful in predicting severe infections requiring amputation.

The estimated normal range of presepsin is less than 300 pg/mL. However, several studies reported the presepsin threshold for sepsis, ranging from 582 to 1025 pg/mL, with a cutoff for sepsis-induced 28- to 30-day mortality spanning 821–1898.5 pg/mL [33,34,35,36,37,38]. A significant threshold variation persists across studies. Although presepsin is recognized as an indicator of sepsis, recent studies have been published exploring its relationship with infectious diseases [39,40,41,42,43,44,45]. The defined cutoff value of 600 pg/mL was established for bacterial infectious diseases [36]. Another study showed that the cutoff value of presepsin was 2080 pg/mL in patients undergoing hemodialysis with skin infections [46]. This study examined the association between presepsin and severe infection requiring amputation in patients with diabetic foot ulcers without sepsis. The findings reveal that an elevated presepsin level is correlated with an increased risk of a severe infection needing amputation, with the established cutoff value being 675 pg/mL. Karakas et al. demonstrated that the soluble CD14 level did not show a significant difference between the amputation group and the non-amputation group in patients with diabetes [47]. However, this study showed a significant difference between the amputation group and non-amputation group.

Presepsin has been found to be significantly elevated in patients with kidney dysfunction [48,49]. In this study, when comparing the eGFR according to the presepsin groups, a trend of a decreasing eGFR with increased presepsin levels was observed. However, in the regression analysis, severe infection requiring amputation and eGFR did not yield statistically significant results.

Poor blood glucose control can be expected to increase the risk of diabetic foot complications, but there was no significant association between HbA1c and diabetic foot amputation in a recent meta-analysis [50]. However, this study found that the risk of severe infection requiring amputation increased as glycated hemoglobin increased. Therefore, the importance of blood glucose control in patients with diabetes can be emphasized once again through this result.

The strength of this study lies in the fact that it is the first study to confirm the relationship between presepsin and diabetic foot ulcers. In addition, it was investigated whether presepsin could serve as an indicator of severe infection requiring amputation in patients with diabetic foot ulcers. Also, it was confirmed that glycated hemoglobin levels were high in patients with severe infection requiring amputation. The limitation of this study includes its small sample size, as it was a retrospective and single-center study. Furthermore, this study relied on the presepsin measurement at the time of hospitalization, so the change in presepsin levels according to the progression of the disease could not be confirmed. An analysis was conducted by determining the cutoff of presepsin, but the significance was not confirmed in the continuous variables. Therefore, it is recommended that further research should encompass large-scale, prospective, or multi-center studies. Additionally, investigating the change in presepsin levels throughout the progression of the disease would be valuable, with a focus on tracking the presepsin level over time.

In conclusion, presepsin, known as an early indicator of sepsis, may serve as a useful marker for severe infection, and it is expected that it can be used for the early prediction of serious infections in diabetic foot ulcerations in the actual clinical field.

## Figures and Tables

**Table 1:** Baseline characteristics.

	No Amputation (n = 27)	Amputation (n = 46)	*p* Value
Age (year)	60.2 ± 18.3	61.8 ± 14.1	0.95
Gender (male %)	12 (44.4%)	32 (69.6%)	0.04
BMI ^1^ (kg/m^2^)	22.4 ± 4.9	24.1 ± 4.2	0.06
Duration of diabetes (year)	19.1 ± 8.8	18.2 ± 9.6	0.69
Hospital stays (day)	21.1 ± 11.4	32.4 ± 18.7	<0.01
HbA1c ^2^ (%)	8.5 ± 2.1	9.2 ± 2.5	0.28
BUN ^3^ (mg/dL)	26.4 ± 14.7	30.5 ± 19.1	0.47
Creatinine (mg/dL)	1.7 ± 1.3	3.0 ± 3.4	0.31
eGFR ^4^ (mL/min/1.73 m^2^)	57.5 ± 34.2	56.1 ± 49.0	0.48
Osteomyelitis (%)	6 (22.2%)	36 (78.3%)	<0.01
Ankle Brachial Index	0.9 ± 0.4	0.9 ± 0.4	0.90
Toe Brachial Index	0.5 ± 0.4	0.4 ± 0.4	0.17
Wound culture (%)	19 (26.0%)	41 (56.2%)	0.05
Texas grade			
Grade 0	0 (0%)	1 (1.4%)	<0.01
Grade 1	15 (20.5%)	10 (13.7%)	
Grade 2	9 (12.3%)	18 (24.7%)	
Grade 3	3 (4.1%)	17 (23.3%)	
Texas stage			
Stage A	6 (8.2%)	6 (8.2%)	0.88
Stage B	6 (8.2%)	18 (24.7%)	
Stage C	7 (9.6%)	7 (9.6%)	
Stage D	8 (11.0%)	15 (20.5%)	
MAC ^5^ score			
No	20 (27.4%)	28 (38.4%)	0.50
Moderate	3 (4.1%)	17 (23.3%)	
Severe	4 (5.4%)	1 (1.4%)	
Smoking (%)	4 (5.5%)	16 (21.9%)	0.07
Drinking (%)	3 (4.1%)	8 (11.0%)	0.47
Hypertension (%)	19 (26.0%)	27 (37.0%)	0.32
Dialysis (%)	2 (2.7%)	12 (16.4%)	0.05
Diabetic retinopathy (%)	4 (5.5%)	13 (17.8%)	0.19
Diabetic neuropathy (%)	2 (2.7%)	5 (6.8%)	0.63
Stroke (%)	4 (5.5%)	4 (5.5%)	0.42
Coronary artery disease (%)	7 (9.6%)	12 (16.4%)	0.99

^1^ Body Mass Index; ^2^ Hemoglobin A1c; ^3^ Blood Urea Nitrogen; ^4^ Estimated Glomerular Filtration Rate; ^5^ Medial Arterial Calcification.

**Table 2:** Characteristics according to cutoff of presepsin for predicting severe infection requiring amputation.

Presepsin (pg/mL)	<675 (n = 33)	≥675 (n = 40)	*p* Value
Presepsin (pg/mL)	428.6 ± 158.7	3402.2 ± 3728.9	<0.01
Age (year)	65.0 ± 15.3	58.0 ± 15.4	0.04
Gender (male %)	19 (26.0%)	25 (34.2%)	0.67
BMI (kg/m^2^)	22.2 ± 5.0	24.5 ± 3.8	0.01
Duration of diabetes (year)	21.7 ± 8.6	15.9 ± 9.0	0.01
Hospital stays (day)	25.0 ± 19.5	30.9 ± 14.8	0.02
Wound culture (%)	27 (37%)	33 (45.2%)	0.94
WBC (K/µL)	9.4 ± 3.5	16.4 ± 8.8	<0.01
ESR (mm/H)	53.8 ± 36.2	89.0 ± 35.2	<0.01
CRP (mg/dL)	4.8 ± 5.5	16.3 ± 11.1	<0.01
Procalcitonin (mg/mL)	0.1 ± 0.2	2.9 ± 4.6	<0.01
HbA1c (%)	8.6 ± 2.3	9.2 ± 2.5	0.23
eGFR (mL/min/1.73 m^2^)	76.9 ± 43.7	39.8 ± 36.7	<0.01
Ankle Brachial Index	18 (24.7%)	24 (32.9%)	0.64
Toe Brachial Index	0.9 ± 0.4	0.9 ± 0.4	0.78
Osteomyelitis (%)	0.5 ± 0.4	0.4 ± 0.4	0.64
Texas grade			
Grade 0	0 (0.0%)	1 (1.4%)	0.24
Grade 1	14 (19.2%)	11 (15.1%)	
Grade 2	12 (16.4%)	15 (20.5%)	
Grade 3	7 (9.6%)	13 (17.8%)	
Texas stage			
Stage A	4 (5.5%)	8 (10.9%)	0.48
Stage B	11 (15.1%)	13 (17.8%)	
Stage C	7 (9.6%)	7 (9.6%)	
Stage D	11 (15.1%)	12 (16.4%)	
Amputation			
No	18 (24.7%)	9 (12.3%)	<0.01
Minor	13 (17.8%)	21 (28.8%)	
Major	2 (2.7%)	10 (13.7%)	
MAC score			
No	26 (35.6%)	22 (30.2%)	0.05
Moderate	5 (6.9%)	15 (20.5%)	
Severe	2 (2.7%)	3 (4.1%)	

**Table 3:** Risk factor assessment of diabetic foot ulcer with severe infection requiring amputation.

	Crude OR ^1^ (95% CI)	Crude *p* Value	Adjusted ^2^ OR (95% CI)	Adjusted *p* Value
Age (year)	1.06 (1.00, 1.13)	0.06	1.06 (1.00, 1.13)	0.05
Gender (male %)	0.68 (0.11, 4.05)	0.67		
WBC (K/µL)	0.79 (0.67, 0.93)	0.01	0.79 (0.67, 0.93)	0.01
ESR (mm/H)	1.03 (0.99, 1.07)	0.13	1.03 (1.00, 1.06)	0.05
CRP ≥ 4.978 (mg/dL)	0.74 (0.05, 11.60)	0.83		
Procalcitonin ≥ 0.186 (mg/mL)	0.60 (0.07, 5.15)	0.64		
Presepsin ≥ 675 (pg/mL)	171.91 (5.12, 5770.37)	<0.01	64.64 (4.14, 1010.23)	<0.01
HbA1c (%)	1.81 (0.96, 3.41)	0.07	1.80 (1.03, 3.12)	0.04
Osteomyelitis (%)	152.67 (10.61, 2196.24)	<0.01	134.99 (10.33, 1763.35)	<0.01
eGFR (mL/min/1.73 m^2^)	1.01 (0.99, 1.04)	0.31		
Wound culture (%)	1.95 (0.12, 30.50)	0.64		

^1^ Odds ratio; ^2^ adjusted by age, WBC, ESR, presepsin ≥ 675, HbA1c, osteomyelitis.

## Data Availability

The data presented in this study are available on request from the corresponding author.
